# Long-Term Persistence of Spike Protein Antibody and Predictive Modeling of Antibody Dynamics After Infection With Severe Acute Respiratory Syndrome Coronavirus 2

**DOI:** 10.1093/cid/ciab607

**Published:** 2021-07-04

**Authors:** Louis Grandjean, Anja Saso, Arturo Torres Ortiz, Tanya Lam, James Hatcher, Rosie Thistlethwayte, Mark Harris, Timothy Best, Marina Johnson, Helen Wagstaffe, Elizabeth Ralph, Annabelle Mai, Caroline Colijn, Judith Breuer, Matthew Buckland, Kimberly Gilmour, David Goldblatt, COVID-19 Staff Testing of Antibody Responses Study (Co-Stars) team, Dorcas Mirambe-Korsah, Fernanda Fenn Torrente, Jakub Wyszynski, Victoria Gander, Amy Leonard, Louise Myers, Aimee Vallot, Camille Paillas, Rose Fitzgerald, Adam Twigg, Rabia Manaf, Lois Gibbons, Hollie Powell, Richard Nar-Dorh, Ally Gray, Elias Fernandez, Aline Minja, Emily Beech, Waffa Girshab, Pei Shi Chia, Kate Webb, Malti Nakrani, Kim Gardiner, Valerija Karaluka, Karen Ryan, Dorothy Lee Katie Groves, Hamad Khan, Shamime Nsubuga, Olivia Rosie-Wilkinson, Julia Spires, Nuria Sanchez-Clemente, Sapriya Kaur, Natasha Carroll, Jemma Efford, Gabriel Bredin, Celma Marisa Dos Santos Domingues, Sophie Foxall, Helen Ashton, Abbey Afzal, Sally Mainland, Kate Crumpler, Lucinda Dawson, Claire Smith, Maria Tabbu, Laura Chiverton, Jade Sugars, Jordan Mooney, Dorothy Chikusu, Fariba Tahami, Baratth Samy, Shomona Begum, Dhimple Patel, Philippa Wiltshire, Annie Susay, Anna Ryan, Luke Lancaster, Kavita Thind, Kate Speller, Rachel Sterling, Connor Tugulu, Sandhya Ghurburrun, Steffi Gray, Joy Mugas, Moe Kishma, Kathleen Akpokomua, Sophie White, Eleana Pieri, Sabina Shamsad, Demi Alexandrou, Odera Aguele, Katherine Miles, Anamika Jain, Subishma Gautam, Oliver Simms, Rachel Goff, Zarif Shams, Tinya Chirinda, Aaliya Nur, Tarekur Rahman

**Affiliations:** 1 Department of Infection, Inflammation and Immunity, Great Ormond Street Institute of Child Health, University College London, London, United Kingdom; 2 Department of Infectious Diseases, Great Ormond Street Hospital, London, United Kingdom; 3 Department of Tropical and Infectious Diseases, London School of Hygiene & Tropical Medicine, London, United Kingdom; 4 MRC Gambia at London School of Hygiene & Tropical Medicine, Fajara, The Gambia; 5 Department of Medicine, Imperial College, Paddington, London, United Kingdom; 6 Department of Microbiology, Great Ormond Street Hospital, London, United Kingdom; 7 Management, Great Ormond Street Hospital, London, United Kingdom; 8 Quality Improvement, Great Ormond Street Hospital, London,United Kingdom; 9 Clinical Immunology, Camelia Botnar Laboratories, Great Ormond Street Hospital, London,United Kingdom; 10 Department of Mathematics, Simon Fraser University, Vancouver, British Colombia, Canada

**Keywords:** immunity, serology, antibody, ELISA, kinetics, neutralization, SARS-CoV-2, COVID-19, virus, nucleoprotein, spike protein

## Abstract

**Background:**

Antibodies to severe acute respiratory syndrome coronavirus 2 (SARS-CoV-2) have been shown to neutralize the virus in vitro and prevent disease in animal challenge models on reexposure. However, the current understanding of SARS-CoV-2 humoral dynamics and longevity is conflicting.

**Methods:**

The COVID-19 Staff Testing of Antibody Responses Study (Co-Stars) prospectively enrolled 3679 healthcare workers to comprehensively characterize the kinetics of SARS-CoV-2 spike protein (S), receptor-binding domain, and nucleoprotein (N) antibodies in parallel. Participants screening seropositive had serial monthly serological testing for a maximum of 7 months with the Meso Scale Discovery Assay. Survival analysis determined the proportion of seroreversion, while 2 hierarchical gamma models predicted the upper and lower bounds of long-term antibody trajectory.

**Results:**

A total of 1163 monthly samples were provided from 349 seropositive participants. At 200 days after symptoms, >95% of participants had detectable S antibodies, compared with 75% with detectable N antibodies. S antibody was predicted to remain detectable in 95% of participants until 465 days (95% confidence interval, 370–575 days) using a “continuous-decay” model and indefinitely using a “decay-to-plateau” model to account for antibody secretion by long-lived plasma cells. S-antibody titers were correlated strongly with surrogate neutralization in vitro (*R*^2^ = 0.72). N antibodies, however, decayed rapidly with a half-life of 60 days (95% confidence interval, 52–68 days).

**Conclusions:**

The Co-Stars data presented here provide evidence for long-term persistence of neutralizing S antibodies. This has important implications for the duration of functional immunity after SARS-CoV-2 infection. In contrast, the rapid decay of N antibodies must be considered in future seroprevalence studies and public health decision-making. This is the first study to establish a mathematical framework capable of predicting long-term humoral dynamics after SARS-CoV-2 infection.

**Clinical Trials Registration:**

NCT04380896.

## Teaser

We demonstrate persistence of spike protein and decay of nucleoprotein antibody in serial samples from 349 patients up to 200 days after severe acute respiratory syndrome coronavirus 2 infection and provide a mathematical modeling framework to predict long-term immune responses.

Since appearing as a cluster of pneumonia cases in Wuhan, China, coronavirus disease 2019 (COVID-19), caused by severe acute respiratory syndrome (SARS) coronavirus (CoV) 2 (SARS-CoV-2), has rapidly spread worldwide [1]. As of 24 April 2021, a total of 145 774 566 cases have been recorded cases globally, resulting in >3 million deaths [2]. Specific immunoglobulin (immunoglobulin [Ig] G) antibody responses to the SARS-CoV-2 trimeric spike (S) protein, nucleoprotein (N), and the receptor-binding domain (RBD) develop 6–15 days after disease onset [3]. The S protein, which contains the RBD, binds to host cells via the angiotensin-converting enzyme 2 (ACE-2) receptor, and membrane fusion occurs before viral entry [4, 5]. Nucleoprotein plays an important role in transcription enhancement and viral assembly [6].

Neutralizing SARS-CoV-2–specific antibodies to the S and RBD antigens, have been shown to be correlated with viral neutralization in vitro as well as to protect against disease in animals after passive transfer of convalescent or monoclonal antibodies [7–11]. It is unclear, however, whether reinfection can occur in humans who mount a humoral response after primary SARS-CoV-2 infection and achieve viral clearance. Neutralizing SARS-CoV antibodies have been shown to commonly persist up to 2–3 years after infection, particularly in hospitalized patients [12, 13], with reports demonstrating seropositivity as long as 12–17 years after infection [14, 15]. Existing longitudinal studies of SARS-CoV-2 are limited by inadequate modeling of antibody dynamics, short duration, low sampling density, and insufficient frequency of follow-up [16–26]. Fitting locally estimated scatterplot smoothing or equivalent lines of best fit to the data also fails to provide a mathematical framework for evaluating long-term antibody responses [16–18, 27].

To evaluate antibody kinetics and longevity after SARS-CoV-2 infection, we undertook the prospective COVID-19 Staff Testing of Antibody Responses Study (Co-Stars). Detailed demographic, clinical and socioeconomic data were collected and mathematical models developed to characterize longitudinal humoral kinetics from initial antibody boosting to subsequent decay. To predict long-term antibody dynamics, we fitted 2 models based on the gamma distribution: one that assumed persistent antibody decay [28] and an alternate that allowed for an eventual plateau, to account for sustained antibody production by long-lived plasma cells [29, 30].

## MATERIALS AND METHODS

### Study Setting and Design

Co-Stars was a 1-year single-center, 2-arm, prospective cohort study of healthcare workers at Great Ormond Street Hospital for Children, London. The study was approved to start by the UK National Health Service Health Research Authority on 29 April 2020 and registered on ClinicalTrials.gov (NCT04380896). Written informed consent was obtained from all participants before recruitment. The Study Protocol and Supplementary Methods and Materials submitted with this article include detailed methods, power calculations. and the data analysis approach.

### Study Participants

All hospital staff members ≥18 years of age were eligible for the study, provided they did not display symptoms consistent with SARS-CoV-2 infection at recruitment. Those who were significantly immunosuppressed or who had previously received blood products (including immunoglobulins or convalescent sera) since September 2019 were excluded from the study.

### Data Collection

Participants undertook a detailed, standardized online questionnaire at study entry. This included sociodemographic factors, details of previous exposure to and symptomatic episodes consistent with COVID-19, any subsequent complications, previous SARS-CoV-2 diagnostic test results, medical and contact history, and a comprehensive assessment of risk factors for exposure, susceptibility to infection, and severe disease. Blood samples were also taken at baseline and each follow-up visit for SARS-CoV-2 serology.

### Polymerase Chain Reaction Measurement of SARS-CoV-2 Serum Antibody and Viral RNA

All 3657 participants underwent a screening enzyme-linked immunosorbent assay (ELISA) with the EDI assay. Those who were identified as seropositive by the EDI assay and provided ≥2 samples had serology repeated by the Meso Scale Discovery (MSD) Chemiluminescent assay that simultaneously detects and quantifies anti–SARS-CoV-2 IgG specific for trimeric S protein, RBD, and N.

### Follow-up Appointments

All seropositive participants were followed up monthly for repeat antibody testing. Seronegative participants were followed up every 6 months. At each follow-up visit, participants completed a shortened version of the baseline questionnaire, focusing on any recurrent COVID-19 exposure and/or symptoms.

### Study Outcomes

The primary outcome of the study was to establish humoral dynamics after SARS-CoV-2 infection. Kaplan-Meier survival analysis was used to compare and plot the time to negativity for each antibody. Two mixed effects gamma models were used to predict the antibody trajectory over time (Supplementary Methods). The “gamma-decay model” hypothesized continuous antibody decay and did not account for long-lived plasma cell antibody production. In contrast, the “gamma-plateau” model allowed for 2 phases of plasma cell production: “short-lived” plasma cells, followed by a subsequent robust long-lived plasma cell response that maintains circulating long-term antibody titers [31].

### Surrogate Neutralization Assay

A 96-well custom competition assay, designed to measure the inhibition of ACE-2 receptor binding to S protein or RBD by serum-derived antibody (MSD), was performed on 94 serial samples from 46 participants (2 participants had 3 serial samples) to establish in vitro correlates of functional immunity.

## RESULTS

### Participant Demographics

A total of 3679 healthcare workers at Great Ormond Street Hospital were enrolled in the study, of whom 733 (19.9%) were SARS-CoV-2 seropositive according to the EDI ELISA. Of these seropositive participants 49% were completely asymptomatic (359 of 733). Of those who were symptomatic, 349 were confirmed seropositive by the MSD assay and provided ≥2 monthly samples for the primary outcome analysis of antibody dynamics (Table 1). This group was followed up monthly for a maximum of 7 months and provided 1163 serial monthly serological samples.

**Table 1.** Demographic Characteristics of Study Participants and Variables Associated With High Peak Antibody Titers

Characteristic or Variable	Participants, No. (%)^a^	Coefficient for Association With Increased S Antibody Titers (*P* Value)
All study recruits, no.	3679	…
Seropositive participants with ≥2 samples	349 (100)	…
Monthly samples, total no.	1163	…
Age, y		
18–30	82 (24)	Reference
30–40	109 (31)	−0.27 (.2)
40–50	83 (24)	−0.15 (.5)
50–60	56 (16)	0.09 (.74)
60–70	19 (5)	0.24 (.5)
Sex		
Female	259 (80%)	−0.3 (.2)
Male	190 (20)	
Profession		
Allied health professionals	83 (24)	Reference
Nurse	101 (29)	0.12 (.53)
Manager	1 (<1)	0.64 (.5)
Cleaner, caterer, or porter	18 (5)	0.4 (.3)
Physician	49 (14)	−0.18 (.5)
Scientist	5 (1)	0.23 (.7)
Symptoms		
Anosmia/ageusia	201 (58)/210 (60)	0.54 (.01)^b^
Cough/shortness of breath/wheezing	225 (64)/130 (37)/67 (19)	0.3 (.14)
Diarrhea/vomiting/diminished appetite	77 (22)/24 (7)/32 (9)	0.3 (.13)
Extreme fatigue/myalgia	199 (57)/225 (64)	−0.08 (.65)
Fever/rigors	175 (50)/27 (8)	0.37 (.03)^b^
Other	174 (50)	Reference
Symptom severity		
Attended hospital	11 (3)	…
Admitted to hospital	1 (0.2)	…
Any comorbid condition		
Yes	42 (13)	0.05 (.8)
No	307 (87)	Reference
Symptom duration, mean (IQR), d	24 (7–27)	0.0007 (.8)
Ethnic background		
BAME	97 (29)	0.42 (.02)^b^
Non-BAME	252 (71)	Reference
Body mass index		
18–25	91	Reference
25–30	42	0.57 (.03)^b^
30–40	34	0.69 (.03)^b^
Missing or unknown	182	NA

Abbreviations: BAME, black, Asian, and minority ethnic; IQR, interquartile range; NA, not applicable; S, spike protein.

^a^Data represent no. (%) of participants unless otherwise specified.

^b^Significant at *P* < .05.

The median follow-up time per participant was 122 days (interquartile range, 65–157 days), with a maximum follow-up time of 262 days from symptom onset. The majority of participants 252 of 349 (72%) donated≥ 3 samples during follow-up, with a maximum of 7 samples. Most seropositive participants (80%) were women, and these participants had a mean age of 39 years, representative of the underlying population structure of the hospital. The predominant symptoms reported were cough 225 of 349 (64%) and myalgia 225 of 349 (64%), followed by ageusia and anosmia in 210 of 349 (60%) and 201 of 349 (58%), respectively.

### Factors Associated With Increased Peak Antibodies and Rapid Decay

Multivariate analysis demonstrated that fever, rigors, ageusia, anosmia, high body mass index, and black, Asian, and minority ethnic backgrounds were all associated with higher peak S-antibody titers (Table 1). No variables were identified as independently associated with the rate of antibody decay.

### Observed Antibody Kinetics and Seroreversion

**Figure 1.**
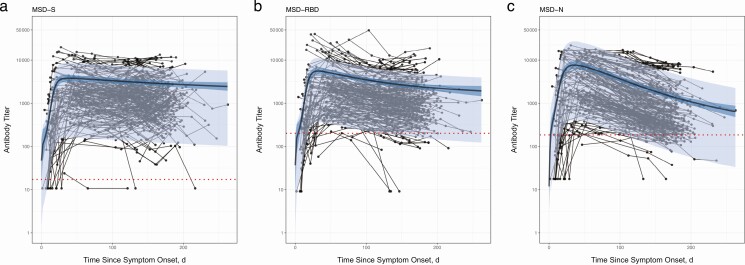
Serial monthly serological measurements from 349 participants up to 262 days after onset of symptoms with severe acute respiratory syndrome coronavirus 2 infection. Samples from the same participant are linked with thin black lines; dotted red lines represent seroreversion. The gamma-plateau model is superimposed to show antibody trajectories; the predicted antibody trajectory (*black line*) is the median of the posterior distribution of the best model fit and 95% confidence interval, with (*light-blue shading*) and without (*dark-blue shading*) individual effects. *A,* Spike protein (S) antibody. *B,* Receptor-binding domain (RBD) antibody. *C,* Nucleoprotein (N) antibody, with a relatively steep rate of decay. Abbreviation: MSD, Meso Scale Discovery assay.

**Figure 2.**
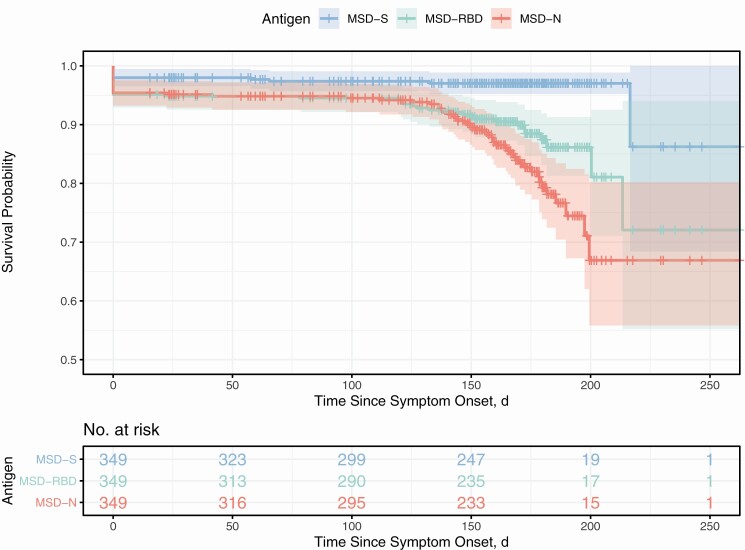
Parallel serological measurements of the spike protein (S; *blue*), receptor-binding domain (RBD; *green*), and nucleoprotein (N; *red*) antibodies from the start of symptoms. Repeated demonstrate the time to a negative test result from all possible starting positive results. The initial number of seropositive participants providing the first sample and the subset still enrolled in the study at any given time point are provided in the table below the graphic. The 95% confidence intervals of this uncertainty are represented by shaded areas around the lines. Abbreviation: MSD, Meso Scale Discovery assay.

Serial monthly serological measurements from 349 participants demonstrated a rapid rate of decay of the N antibody relative to the S and RBD antibodies (Figure 1). The S-antibody assay identified 342 of 349 participants (98%) who were seropositive to any of the S, RBD, or N antibodies. In comparison the RBD and N assays identified 332 (95%) and 333 (95%) who were seropositive, respectively. The sensitivity of the RBD- and N-antibody assays further declined with time relative to the S-antibody assay. At 150 days after infection 249 of the 349 participants initially seropositive for the N antibody provided samples for analysis. Only 233 of 249 remained positive to the N antibody (survival probability 95% confidence interval [CI], .86–.93) while significantly more, 247 of 249 (.95–.99) remained positive to the S antibody. At 200 days after infection, 19 samples were available for comparison, of which 15 remained positive to the N antibody (survival probability 95% CI, .56–.80) while 19 remained positive to the S antibody (.95–.99, Figure 2).

### Modeled Serological Reversion and Proportion of Positive Test Results Over Time

**Figure 3.**
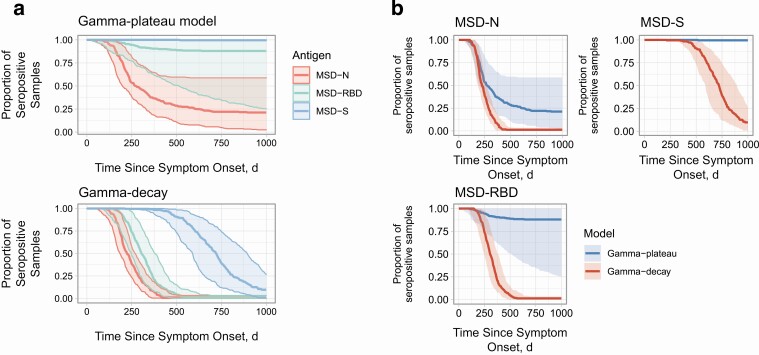
Model-based predictions of time to seronegativity from symptom onset. *A,* Comparison of the 3 tested antibodies against the spike protein (S; *blue*), receptor-binding domain (RBD; *green*), and nucleoprotein (N; *red*) for the gamma-plateau (top) and the gamma-decay (*bottom*) models. *B,* Differences between the gamma-plateau (*blue*) and gamma-decay (*red*) models for the 3 tested antibodies: S (*top right*), RBD (*bottom*), and N (*top left).* Colored lines represent median estimates of the posterior density, while the shaded ribbons represent 95% confidence intervals. Abbreviation: MSD, Meso Scale Discovery assay.

Comparison of goodness of fit between models showed that for all antigens the decay-to-plateau model provided a better fit to the data than the gamma-decay model, although this difference was not statistically significant (Supplementary Table 2). Even under the most pessimistic assumption of continuous gamma decay we estimate that 95% of individuals after infection with SARS-CoV-2 will have measurable S antibody until 465 days (95% CI, 370–575 days) after symptom onset. Under the gamma-plateau model, S antibody is predicted to remain detectable indefinitely (Figure 3A) [15]. The most pessimistic gamma-decay model (lower bound) and most optimistic gamma-plateau model (upper bound) for each antibody are shown in Figure 3B. Under both models the N antibody decayed to undetectable levels. Even with the gamma-plateau model, 75% of participants were predicted to have seroreverted N antibody by 610 (95% CI, 420–530) days after symptom onset, whereas with the gamma-decay model 100% of participants had seroreverted N antibody by 460 (420–530) days . Fewer serial samples beyond 200 days after symptom onset increased the uncertainty in our longer-term modeled estimates of antibody duration (shaded areas in Figure 3).

### Antibody Peak, Half-Life, and Plateau

**Figure 4.**
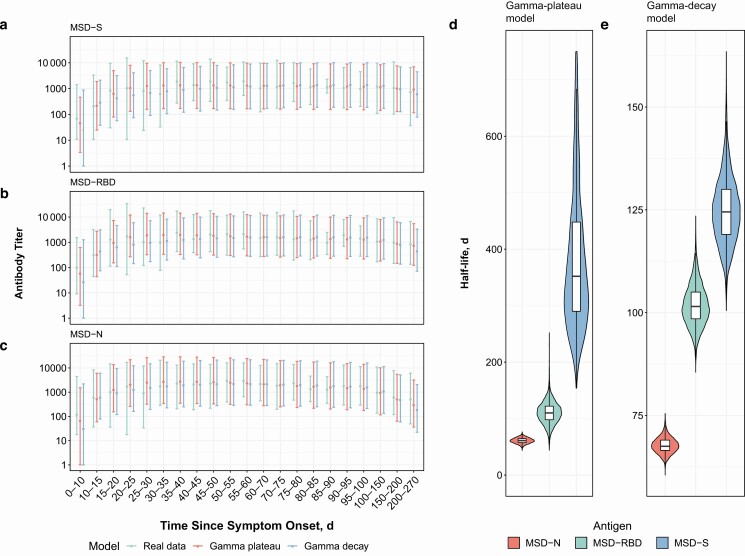
Measured and modeled weekly mean antibody titers. *A–C,* Real data (*green*), gamma-plateau model (*red*), and gamma-decay model (*blue*) for the spike protein (S [*A*]), receptor-binding domain (RBD [*B*]), and nucleoprotein (N [*C*]) antibodies. *D, E,* Modeled half-lives of antibody decay with the gamma-plateau (*D*) and gamma-decay (*E*) models for S (*blue*), N (*red*), and RBD (*green*) antibodies. Abbreviation: MSD, Meso Scale Discovery assay.

Antibody titers rapidly increased during the first 3 weeks, with prolonged high titers reached and maintained between weeks 4 and 10 after symptom onset. The peak antibody responses for the S, RBD, and N antibodies from both raw weekly average serial titer and modeled data occurred at 40 (95% CI, 30–63), 31 (26–38), and 35 (31–42) days, respectively. This finding was supported by the both the gamma-decay and gamma-plateau models, which provided a similar close fit to this early stage of the humoral response (Figure 4A–C).

The modeled half-lives under the gamma-decay and gamma-plateau models were also very similar, and both models showed rapid decay of the N antibody relative to the RBD and S antibodies. The half-lives for the N, RBD, and S antibodies were 60 (95% CI, 52–68), 102 (92–114), and 126 (112–146) days, respectively, compared with 60 (52–70), 110 (74–148), and 364 (212–997) days for the gamma-plateau model (Figure 4D and 4E).

Under the gamma-plateau model, the S antibody was predicted to decay slowly, reaching an eventual stabilized plateau at 1825 days since symptom onset (95% CI, 250–3700 days), at which point the titer still remained above the threshold for a negative test; the N antibody, on the other hand, was predicted to decay to a plateau by 610 days, crossing the threshold for a negative test.

### Surrogate Neutralization Assay

**Figure 5.**
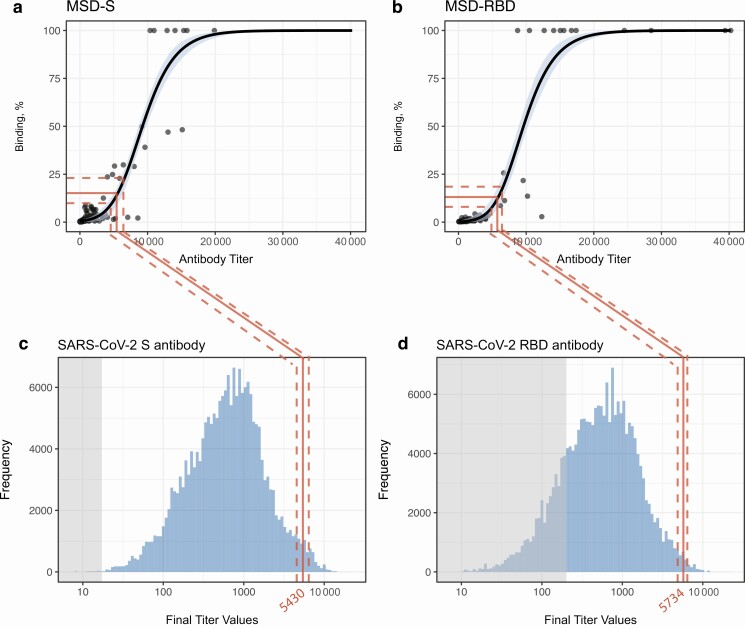
Surrogate neutralization assay (spike protein [S] and receptor-binding domain [RBD]), with mapping to the final predicted titers at plateau. *A, B,* Percentage of binding plotted against antibody titers. Red lines represent the median antibody titer at which the change in percentage binding is greatest; dotted red lines, 95% confidence intervals (CIs). Black lines represent the median posterior distribution of the generalized logistic model, and blue shading, 95% CIs. *C, D,* Posterior distribution of antibody titers at the long-term plateau, as predicted by the gamma-plateau model; shaded gray areas represent thresholds of seronegativity. Abbreviations: MSD, Meso Scale Discovery assay; SARS-CoV-2, severe acute respiratory syndrome coronavirus 2.

There was a sigmoidal relationship between raw antibody titers and percentage binding/ACE-2 receptor blocking for both the S and RBD antibodies. Above a threshold S-antibody titer of 8586 (95% CI, 8160–9095), there was a dramatic increase in percentage binding/ACE-2 receptor blocking. Log-transformed antibody titers were strongly positively correlated with receptor blocking (*R*^2^ = 0.72 for S and *R*^2^ = 0.77 for RBD antibodies). We mapped the point of greatest change in neutralization and the lower limit of detection to the final predicted antibody titers at the plateau (Figure 5). While the full range of the distribution of S antibodies was predicted to remain detectable indefinitely under the gamma-plateau model, only a small proportion of individuals were predicted to have titers sufficient to enable measurable functional binding with our surrogate neutralization assay.

## DISCUSSION

This prospective cohort study of antibody responses after symptomatic SARS-CoV-2 infection has demonstrated that >95% of healthcare workers had persistent detectable S antibodies up to 200 days after infection. Our study is the first to provide a mathematical modeling framework capable of predicting the long-term dynamics of the 3 key SARS-CoV-2 antibodies after natural infection. Even under our most pessimistic assumptions of continuous exponential decay, 95% of individuals were predicted to remain seropositive to S antibody at 465 days (95% CI, 370–575 days) while our more optimistic upper bound gamma-decay model predicted a permanent long-lasting plateau of detectable S antibody.

These data contradict conclusions from studies that have reported rapid waning of antibodies after a few months [22, 24, 25]. Our findings are consistent with the duration of humoral responses observed after SARS-CoV and Middle East respiratory syndrome infections; however, modeling of the S-antibody trajectory under the MSD assay suggests that in the longer term there is a potential to lose neutralizing capability despite having detectable IgG [12–15]. Importantly, the long-lasting S and RBD antibodies are also well correlated with a surrogate SARS-CoV-2 neutralization assay of ACE-2 receptor blocking, strongly suggesting that long-term measurable S-antibody levels are functionally important.

In contrast to the S antibody, the N antibody was observed to serorevert in 56 of 349 participants over the course of the study alone and had a modeled half-life of 60 days. This has important implications for diagnostic testing, epidemiological modeling, and public health decision making, which often rely on the N antibody to estimate SARS-CoV-2 seroprevalence. This finding may also explain some unexpectedly low estimates of population prevalence in high-burden countries [32].

The persistence of detectable S and/or RBD antibody compared with the rapid decay of the N antibody has also been observed in convalescent serum samples obtained from SARS survivors, 17 years after infection [14]. The mechanisms underlying this observation warrant further investigation. Differences in the epitope structure [33], immunogenicity, and presentation to B cells may affect the production, maturation, and longevity of the plasma cells that secrete these antibodies [34–37]. Distinct T-helper cell interactions at the germinal center may further determine B-cell and humoral dynamics, as previously observed in the context of the response to different HIV proteins [38]. The nucleoprotein is more conserved across CoVs than RBD, potentially leading to cross-reactive memory responses, with differing kinetics and less contribution from naive B cells [39].

To date, no studies have comprehensively modeled the nature and duration of antibody responses to different SARS-CoV-2 epitopes. Long et al [19], Seow et al [22] and Ibarrondo et al [20] demonstrated rapid decay of SARS-CoV-2 antibodies within the first 3 months after infection, particularly in mildly symptomatic cases. In comparison, others have reported that the S and/or RBD antibodies are correlated with neutralizing responses and decay slowly, persisting during the study period, up to 90–150 days after infection [16–18, 40]. These studies, however, are limited by their shorter sampling time frame, lower sampling density, and lack of appropriate modeling to predict antibody trajectory. Implementing locally estimated scatterplot smoothing lines of best fit to the data [16–18, 22] or comparing the variance of average antibody titers at different time intervals [24, 25, 40] does not permit evaluation of long-term antibody trajectories.

Our study is strengthened by the density, frequency, and duration of sampling collection. The parallel evaluation of antibody titers to 3 major SARS-CoV-2 proteins by the chemiluminescent MSD assay also enabled us to demonstrate the decay of the N antibody relative to the S and RBD antibodies. Importantly, this is the first study to provide a mathematical framework for long-term SARS-CoV-2 antibody responses, modeling both the peak and decay after infection and enabling realistic best-case and worst-case predictions, as well as considering the impact of long-lived plasma cells on future antibody titers. Our work provides a detailed, shareable, and reproducible model, with parameters that are useful for epidemiological purposes.

None of the seropositive healthcare workers identified in this study required hospitalization. Given that the majority of COVID-19 cases are managed in the community, our study cohort is therefore representative of most SARS-CoV-2 infections in the general population [41]. Severe disease, however, has been associated with higher SARS-CoV-2 antibody titers and potentially a longer-lasting humoral response. Studies in recent years have also hypothesized that previous exposure to seasonal CoVs—to which pediatric healthcare workers may be disproportionately exposed—may confer some protection against SARS-CoV-2 [11, 14, 42–47] and may therefore need to be accounted for when modeling transmission or longevity dynamics [48]. However, previously published work from our group has demonstrated that those with previous exposure to seasonal coronavirus demonstrated little SARS-CoV-2 pseudo-neutralizing activity, thereby limiting its impact on our findings [49].

Furthermore, only 38% of participants in the study had an available confirmatory positive polymerase chain reaction result. To mitigate this concern, a formal evaluation of the MSD assay was undertaken before the beginning of the study, using 169 SARS-CoV-2 polymerase chain reaction–positive participants; 97.9% sensitivity and 97.4% specificity were demonstrated at 21 days after infection [49]. This makes the proportion of false-positive serological tests likely to be small and therefore to have little impact on our findings. The use of a screening ELISA before the chemiluminescent MSD assay may also have resulted in some participants being incorrectly classified as seronegative, particularly those with low-level detectable N-antibodies. A more sensitive screening test, however, would likely lead to an earlier time-to-negativity N trajectory, thereby reinforcing our main findings. Limitations in sample size >200 days after infection also increased our time to negativity and modeling uncertainty. Our estimates of the time to negativity are dependent on the negative thresholds and lower limits of detection of the assay. However, our model fits, as well as estimates of antibody decay and titers (Figure 1), are not dependent on the assay’s lower limits of detection.

No definitive quantitative or qualitative correlate of protection has been identified yet for SARS-CoV-2 infection, disease, or onward transmission. Nevertheless, live viral neutralization assays remain the reference standard in vitro correlate of protection against viral infection; as such, lack of formal “authentic” neutralization tests is a study limitation. However, ACE-2 receptor competition assays, such as the MSD assay we used, have been shown to correlate well with formal viral neutralization assays, enabling use as suitable surrogate functional tests [50]. Recent studies have highlighted the potential for SARS-CoV-2 to gain entry to epithelial cells via CD147 receptor [51]. Blocking of this receptor was not quantified by our competition assay; whether this influences the correlation with in vivo neutralization is unknown.

Finally, it remains to be seen to what extent and at what threshold long-term detectable antibodies induce sterilizing immunity, limit transmission, or simply attenuate disease severity. Human reinfection studies and plaque reduction assays >1 year after infection are required to clarify this further. SARS-CoV-2–specific T and B memory cellular responses must also be characterized to accurately determine durability of immunity. Similarly, mucosal antibody responses may play an important role in the overall protective immune response, particularly in early infection. The neutralizing capability and duration of mucosal IgA responses are currently being studied from the same cohort.

In summary, this prospective cohort study has demonstrated the persistence of SARS-CoV-2 S antibody in >95% of individuals up to 200 days after infection. Our lowest-bound continuous-decay model predicted that 95% of individuals would continue to have detectable S antibody at 465 days, while our upper-bound gamma-plateau model predicted that the S antibody would plateau at detectable levels indefinitely. The long-term presence of functional S (and RBD) antibody has important implications for the duration of protective immunity after natural infection. It remains to be seen whether novel SARS-CoV-2 vaccine candidates will replicate the long S-antibody duration induced by natural infection.

## Supplementary Data

Supplementary materials are available at *Clinical Infectious Diseases* online. Consisting of data provided by the authors to benefit the reader, the posted materials are not copyedited and are the sole responsibility of the authors, so questions or comments should be addressed to the corresponding author.

## Supplementary Material

ciab607_suppl_Supplementary_Figures

ciab607_suppl_Supplementary_Materials_Protocol

ciab607_suppl_Supplementary_Methods

ciab607_suppl_Supplementary_Tables

## Notes

***Author contributions.*** The first draft of the manuscript was prepared by L. G., A. S., A. T. O., T. L., J. H., R. T., M. H., T. B., M. J., H. W., E. R., A. M., C. C., J. B., M. B., K. G., and D. G. The study was designed and implemented by L. G., A. S., A. T. O., T. L., J. H., R. T., M. H., T. B., M. J., H. W., E. R., A. M., C. C., J. B., M. B., K. G., D. G., and the Co-Stars team. Testing for severe acute respiratory syndrome coronavirus 2 RNA and antibody was conducted by L. G., A .S., A. T. O., T. L., J. H., R. T., M .H.., T. B., M. J., H. W., E. R., A. M., C. C., J. B., M. B., K. G., and D. G.

***Acknowledgments.*** The authors dedicate this article to the staff members who died of coronavirus disease 2019 at Great Ormond Street Hospital during the first wave of the pandemic. They also thank all staff at Great Ormond Street Hospital who have taken part in the study. In addition, the authors are very grateful for all the hard work undertaken by the Great Ormond Street laboratory staff and the staff in the immunology laboratories both in the Camelia Botnar Laboratory and the Great Ormond Street Institute of Child Health, who ensured that all polymerase chain reaction tests and serological assays were completed in a timely manner. Finally, they acknowledge the support of the Great Ormond Street Hospital Research & Development, Governance, Finance, Management, Estates, Operations and Communications departments.

***Disclaimer.*** The funders had no role in the study design, data collection and analysis, decision to publish, or preparation of the manuscript.

***Financial support.*** This work was supported by Wellcome Trust (grants 201470/Z/16/Z to L. G. and 220565/Z/20/Z to A. S.), the National Institute of Allergy and Infectious Diseases, National Institutes of Health (grant 1R01AI146338 to L. G.), the Great Ormond Street Hospital (GOSH) Charity (grant VC0921 to L. G.), and the GOSH/Institute of Child Health Biomedical Research Centre (www.nihr.ac.uk; L. G.).

***Potential conflicts of interest.*** The authors: No reported conflicts of interest. All authors have submitted the ICMJE Form for Disclosure of Potential Conflicts of Interest. Conflicts that the editors consider relevant to the content of the manuscript have been disclosed.

***Study group.*** The COVID-19 Staff Testing of Antibody Responses Study (Co-Stars) team includes the following: Dorcas Mirambe-Korsah, Fernanda Fenn Torrente, Jakub Wyszynski, Victoria Gander, Amy Leonard, Louise Myers, Aimee Vallot, Camille Paillas, Rose Fitzgerald, Adam Twigg, Rabia Manaf, Lois Gibbons, Hollie Powell, Richard Nar-Dorh, Ally Gray, Elias Fernandez, Aline Minja, Emily Beech, Waffa Girshab, Pei Shi Chia, Kate Webb, Malti Nakrani, Kim Gardiner, Valerija Karaluka, Karen Ryan, Dorothy Lee Katie Groves, Hamad Khan, Shamime Nsubuga, Olivia Rosie-Wilkinson, Julia Spires, Nuria Sanchez-Clemente, Sapriya Kaur, Natasha Carroll, Jemma Efford, Gabriel Bredin, Celma Marisa Dos Santos Domingues, Sophie Foxall, Helen Ashton, Abbey Afzal, Sally Mainland, Kate Crumpler, Lucinda Dawson, Claire Smith, Maria Tabbu, Laura Chiverton, Jade Sugars, Jordan Mooney, Dorothy Chikusu, Fariba Tahami, Baratth Samy, Shomona Begum, Dhimple Patel, Philippa Wiltshire, Annie Susay, Anna Ryan, Luke Lancaster, Kavita Thind, Kate Speller, Rachel Sterling, Connor Tugulu, Sandhya Ghurburrun, Steffi Gray, Joy Mugas, Moe Kishma, Kathleen Akpokomua, Sophie White, Eleana Pieri, Sabina Shamsad, Demi Alexandrou, Odera Aguele, Katherine Miles, Anamika Jain, Subishma Gautam, Oliver Simms, Rachel Goff, Zarif Shams, Tinya Chirinda, Aaliya Nur, and Tarekur Rahman.

## References

[1] Guan W , Ni Z, Hu Y, et al. Clinical characteristics of coronavirus disease 2019 in China. N Engl J Med 2020; 382:1708–20. 32109013 10.1056/NEJMoa2002032 PMC7092819

[2] Dong E , Du H, Gardner L. An interactive web-based dashboard to track COVID-19 in real time. Lancet Infect Dis 2020; 20:533–4. 10.1016/S1473-3099(20)30120-1 PMC7159018 32087114

[3] Immune responses and immunity to SARS-CoV-2. Available at: https://www.ecdc.europa.eu/en/covid-19/latest-evidence/immune-responses. Accessed 30 October 2020.

[4] Walls AC , Park YJ, Tortorici MA, Wall A, McGuire AT, Veesler D. Structure, function, and antigenicity of the SARS-CoV-2 spike glycoprotein. Cell 2020; 181:281–92.e6. 32155444 10.1016/j.cell.2020.02.058 PMC7102599

[5] Ou X , Liu Y, Lei X, et al. Characterization of spike glycoprotein of SARS-CoV-2 on virus entry and its immune cross-reactivity with SARS-CoV. Nat Commun 2020; 11:1620. 10.1038/s41467-020-15562-9 PMC7100515 32221306

[6] Cong Y , Ulasli M, Schepers H, et al. Nucleocapsid protein recruitment to replication-transcription complexes plays a crucial role in coronaviral life cycle. J Virol 2020; 94:e01925-19. 31776274 10.1128/JVI.01925-19 PMC6997762

[7] Ju B , Zhang Q, Ge J, et al. Human neutralizing antibodies elicited by SARS-CoV-2 infection. Nature 2020; 584:115–9. 32454513 10.1038/s41586-020-2380-z

[8] Imai M , Iwatsuki-Horimoto K, Hatta M, et al. Syrian hamsters as a small animal model for SARS-CoV-2 infection and countermeasure development. Proc Natl Acad Sci U S A 2020; 117:16587–95. 32571934 10.1073/pnas.2009799117 PMC7368255

[9] Rogers TF , Zhao F, Huang D, et al. Isolation of potent SARS-CoV-2 neutralizing antibodies and protection from disease in a small animal model. Science 2020; 369:956–63. 32540903 10.1126/science.abc7520 PMC7299280

[10] Suthar MS , Zimmerman MG, Kauffman RC, et al. Rapid generation of neutralizing antibody responses in COVID-19 patients. Cell Rep Med 2020; 1:100040. 32835303 10.1016/j.xcrm.2020.100040 PMC7276302

[11] Ni L , Ye F, Cheng ML, et al. Detection of SARS-CoV-2-specific humoral and cellular immunity in COVID-19 convalescent individuals. Immunity 2020; 52:971–7.e3. 32413330 10.1016/j.immuni.2020.04.023 PMC7196424

[12] Cao WC , Liu W, Zhang PH, Zhang F, Richardus JH. Disappearance of antibodies to SARS-associated coronavirus after recovery. N Engl J Med 2007; 357:1162–3. 17855683 10.1056/NEJMc070348

[13] Mo H , Zeng G, Ren X, et al. Longitudinal profile of antibodies against SARS-coronavirus in SARS patients and their clinical significance. Respirology 2006; 11:49–53. 16423201 10.1111/j.1440-1843.2006.00783.x PMC7192223

[14] Chia WN , Tan CW, Foo R, et al. Serological differentiation between COVID-19 and SARS infections. Emerg Microbes Infect 2020; 9:1497–505. 32529906 10.1080/22221751.2020.1780951 PMC7473126

[15] Guo X , Guo Z, Duan C, et al. Long-term persistence of IgG antibodies in SARS-CoV infected healthcare workers. medRxiv [Preprint: not peer reviewed]. February 14, 2020. Available from: https://www.medrxiv.org/content/10.1101/2020.02.12.20021386v1.

[16] Isho B , Abe KT, Zuo M, et al. Persistence of serum and saliva antibody responses to SARS-CoV-2 spike antigens in COVID-19 patients. Sci Immunol 2020; 5:eabe5511. 33033173 10.1126/sciimmunol.abe5511 PMC8050884

[17] Iyer AS , Jones FK, Nodoushani A, et al. Persistence and decay of human antibody responses to the receptor binding domain of SARS-CoV-2 spike protein in COVID-19 patients. Sci Immunol 2020; 5:eabe0367. 33033172 10.1126/sciimmunol.abe0367 PMC7857394

[18] Ripperger TJ , Uhrlaub JL, Watanabe M, et al. Orthogonal SARS-CoV-2 serological assays enable surveillance of low-prevalence communities and reveal durable humoral immunity. Immunity 2020; 53:925–33.e4. 33129373 10.1016/j.immuni.2020.10.004 PMC7554472

[19] Long QX , Tang XJ, Shi QL, et al. Clinical and immunological assessment of asymptomatic SARS-CoV-2 infections. Nat Med 2020; 26:1200–4. 32555424 10.1038/s41591-020-0965-6

[20] Ibarrondo FJ , Fulcher JA, Goodman-Meza D, et al. Rapid decay of anti–SARS-CoV-2 antibodies in persons with mild Covid-19. N Engl J Med 2020; 383:1085–7. 32706954 10.1056/NEJMc2025179 PMC7397184

[21] Christiane M , Johannes CF. Loss of anti–SARS-CoV-2 antibodies in mild Covid-19. N Engl J Med 2020; 383:1694–8. 10.1056/NEJMc2027051 32966710

[22] Seow J , Graham C, Merrick B, et al. Longitudinal evaluation and decline of antibody responses in the three months following SARS-CoV-2 infection in humans. Nat Microbiol 2020; 5:1598–607. 33106674 10.1038/s41564-020-00813-8 PMC7610833

[23] Wajnberg A , Amanat F, Firpo A, et al. SARS-CoV-2 infection induces robust, neutralizing antibody responses that are stable for at least three months. medRxiv [Preprint: not peer reviewed]. October 13, 2020. Available from: https://www.medrxiv.org/content/10.1101/2020.07.14.20151126v1.

[24] Crawford KHD , Dingens AS, Eguia R, et al. Dynamics of neutralizing antibody titers in the months after severe acute respiratory syndrome coronavirus 2 infection. J Infect Dis 2021; 223:197–205. 33535236 10.1093/infdis/jiaa618 PMC7543487

[25] Tan Y , Liu F, Xu X, et al. Durability of neutralizing antibodies and T-cell response post SARS-CoV-2 infection. Front Med 2020; 14:746–51. 33017040 10.1007/s11684-020-0822-5 PMC7533664

[26] Piccoli L , Park YJ, Tortorici MA, et al. Mapping neutralizing and immunodominant sites on the SARS-CoV-2 spike receptor-binding domain by structure-guided high-resolution serology. Cell 2020; 183:1024–42.e21. 32991844 10.1016/j.cell.2020.09.037 PMC7494283

[27] Wu J , Liang B, Chen C, et al. SARS-CoV-2 infection induces sustained humoral immune responses in convalescent patients following symptomatic COVID-19. Nat Commun 2021; 12:1813 . 33753738 10.1038/s41467-021-22034-1 PMC7985370

[28] Zhao X , Ning Y, Chen MIC, Cook AR. Individual and population trajectories of influenza antibody titers over multiple seasons in a tropical country. Am J Epidemiol 2018; 187:135–43. 29309522 10.1093/aje/kwx201 PMC5860523

[29] Fraser C , Tomassini JE, Xi L, et al. Modeling the long-term antibody response of a human papillomavirus (HPV) virus-like particle (VLP) type 16 prophylactic vaccine. Vaccine 2007; 25:4324–33. 17445955 10.1016/j.vaccine.2007.02.069

[30] Andraud M , Lejeune O, Musoro JZ, Ogunjimi B, Beutels P, Hens N. Living on three time scales: the dynamics of plasma cell and antibody populations illustrated for hepatitis A virus. PLOS Comput Biol 2012; 8:e1002418. 22396639 10.1371/journal.pcbi.1002418 PMC3291529

[31] Cyster JG , Allen CDC. B cell responses: cell interaction dynamics and decisions. Cell 2019; 177:524–40. 31002794 10.1016/j.cell.2019.03.016 PMC6538279

[32] Pollán M , Pérez-Gómez B, Pastor-Barriuso R, et al. Prevalence of SARS-CoV-2 in Spain (ENE-COVID): a nationwide, population-based seroepidemiological study. Lancet 2020; 396:535–44. 32645347 10.1016/S0140-6736(20)31483-5 PMC7336131

[33] Slifka MK , Amanna IJ. Role of multivalency and antigenic threshold in generating protective antibody responses. Front Immunol 2019; 10:956. 31118935 10.3389/fimmu.2019.00956 PMC6504826

[34] Dörner T , Radbruch A. Antibodies and B cell memory in viral immunity. Immunity 2007; 27:384–92. 17892847 10.1016/j.immuni.2007.09.002

[35] Amanna IJ , Slifka MK. Mechanisms that determine plasma cell lifespan and the duration of humoral immunity. Immunol Rev 2010; 236:125–38. 20636813 10.1111/j.1600-065X.2010.00912.x PMC7165522

[36] Khodadadi L , Cheng Q, Radbruch A, Hiepe F. The maintenance of memory plasma cells. Front Immunol 2019; 10:721. 31024553 10.3389/fimmu.2019.00721 PMC6464033

[37] Nguyen DC , Joyner CJ, Sanz I, Lee FEH. Factors affecting early antibody secreting cell maturation into long-lived plasma cells. Front Immunol 2019; 10:2138. 31572364 10.3389/fimmu.2019.02138 PMC6749102

[38] Bonsignori M , Moody MA, Parks RJ, et al. HIV-1 envelope induces memory B cell responses that correlate with plasma antibody levels after envelope gp120 protein vaccination or HIV-1 infection. J Immunol 2009; 183:2708–17. 19625640 10.4049/jimmunol.0901068 PMC3089979

[39] Srinivasan S , Cui H, Gao Z, et al. Structural genomics of SARS-CoV-2 indicates evolutionary conserved functional regions of viral proteins. Viruses 2020; 12:360. 10.3390/v12040360 PMC7232164 32218151

[40] Wajnberg A , Amanat F, Firpo A, et al. Robust neutralizing antibodies to SARS-CoV-2 infection persist for months. Science 2020; 370:1227–30. 33115920 10.1126/science.abd7728 PMC7810037

[41] Verity R , Okell LC, Dorigatti I, et al. Estimates of the severity of coronavirus disease 2019: a model-based analysis. Lancet Infect Dis 2020; 20:669–77. 10.1016/S1473-3099(20)30243-7 PMC7158570 32240634

[42] Wang C , Li W, Drabek D, et al. A human monoclonal antibody blocking SARS-CoV-2 infection. Nat Commun 2020; 11:2251. 32366817 10.1038/s41467-020-16256-y PMC7198537

[43] Grifoni A , Weiskopf D, Ramirez SI, et al. Targets of T cell responses to SARS-CoV-2 coronavirus in humans with COVID-19 disease and unexposed individuals. Cell 2020; 181:1489–501.e15. 32473127 10.1016/j.cell.2020.05.015 PMC7237901

[44] Le Bert N , Tan AT, Kunasegaran K, et al. SARS-CoV-2-specific T cell immunity in cases of COVID-19 and SARS, and uninfected controls. Nature 2020; 584:457–62. 32668444 10.1038/s41586-020-2550-z

[45] Juno JA , Tan HX, Lee WS, et al. Humoral and circulating follicular helper T cell responses in recovered patients with COVID-19. Nat Med 2020; 26: 1428–34. 32661393 10.1038/s41591-020-0995-0

[46] Peng Y , Mentzer AJ, Liu G, et al; Oxford Immunology Network Covid-19 Response T cell Consortium; ISARIC4C Investigators. Broad and strong memory CD4^+^ and CD8^+^ T cells induced by SARS-CoV-2 in UK convalescent individuals following COVID-19. Nat Immunol 2020; 21:1336–45. 32887977 10.1038/s41590-020-0782-6 PMC7611020

[47] Hicks J , Klumpp-Thomas C, Kalish H, et al. Serologic cross-reactivity of SARS-CoV-2 with endemic and seasonal betacoronaviruses. J Clin Immunol 2021; 41:906–13. 33725211 10.1007/s10875-021-00997-6 PMC7962425

[48] Kissler SM , Tedijanto C, Goldstein E, Grad YH, Lipsitch M. Projecting the transmission dynamics of SARS-CoV-2 through the postpandemic period. Science 2020; 368:860–8. 32291278 10.1126/science.abb5793 PMC7164482

[49] Johnson M , Wagstaffe HR, Gilmour KC, et al. Evaluation of a novel multiplexed assay for determining IgG levels and functional activity to SARS-CoV-2. J Clin Virol 2020; 130:104572. 32769024 10.1016/j.jcv.2020.104572 PMC7396134

[50] Walker SN , Chokkalingam N, Reuschel EL, et al. SARS-CoV-2 assays to detect functional antibody responses that block ACE2 recognition in vaccinated animals and infected patients. J Clin Microbiol 2020; 58:e01533-20. 32855181 10.1128/JCM.01533-20 PMC7587116

[51] Seyedpour S , Khodaei B, Loghman AH, et al. Targeted therapy strategies against SARS-CoV-2 cell entry mechanisms: a systematic review of in vitro and in vivo studies. J Cell Physiol 2021; 236:2364–92. 32901936 10.1002/jcp.30032

