# Supplementary material for: Long-Term Persistence of Spike Protein Antibody and Predictive Modeling of Antibody Dynamics After Infection With Severe Acute Respiratory Syndrome Coronavirus 2
Source: Clin Infect Dis. 2021 Jul 4;74(7):1220–9. doi: 10.1093/cid/ciab607 (PMC8994590; doi:10.1093/cid/ciab607)
Supplement: ciab607_suppl_Supplementary_Figures [file ciab607_suppl_supplementary_figures.pdf]

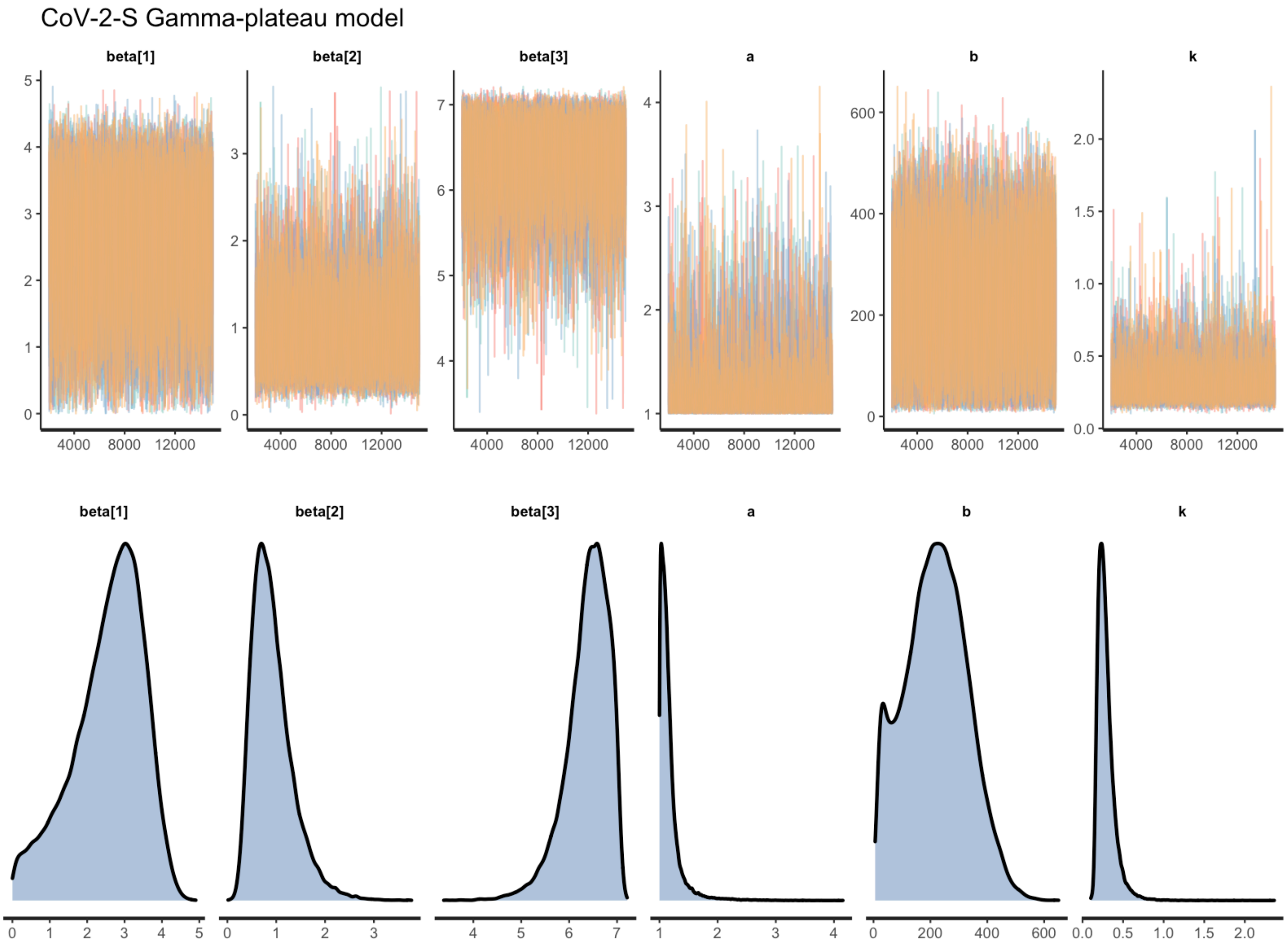
CoV-2-S Gamma-plateau model
beta[1]
beta[2]
beta[3]
a
b
k
4000
8000
12000

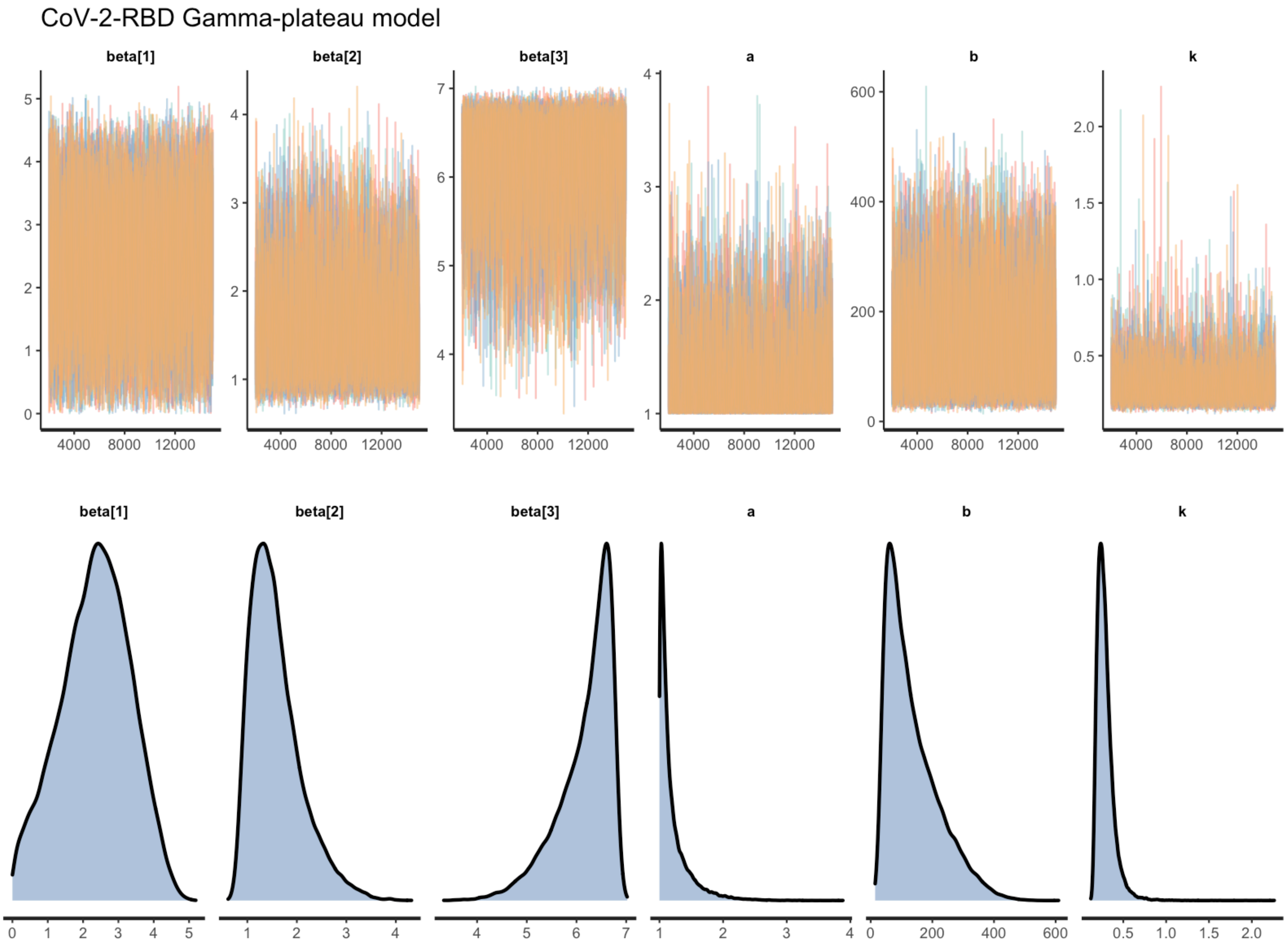

CoV-2-RBD Gamma-plateau model
beta[1]
beta[2]
beta[3]
a
b
k
4000
8000
12000

CoV-2-N Gamma-plateau model

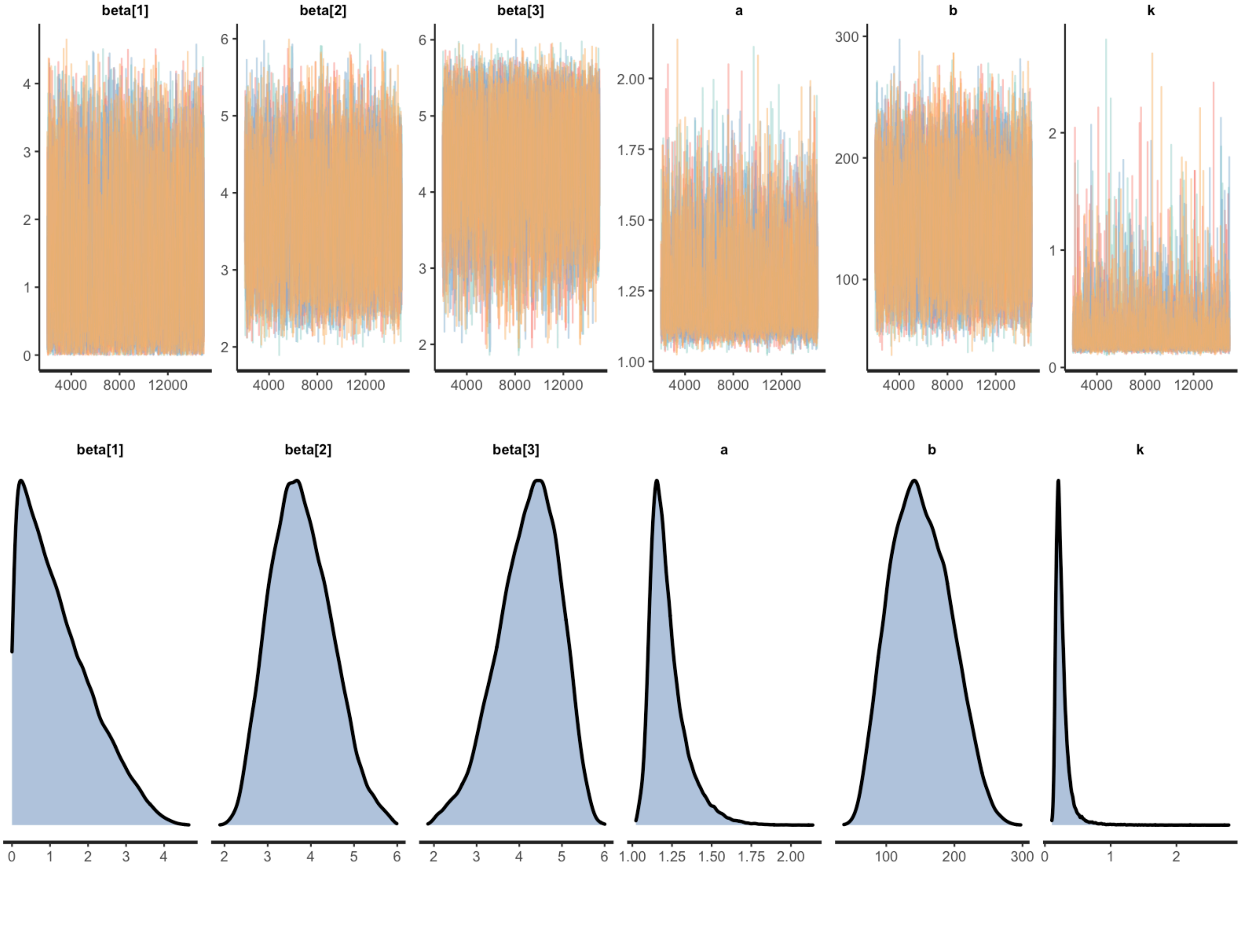

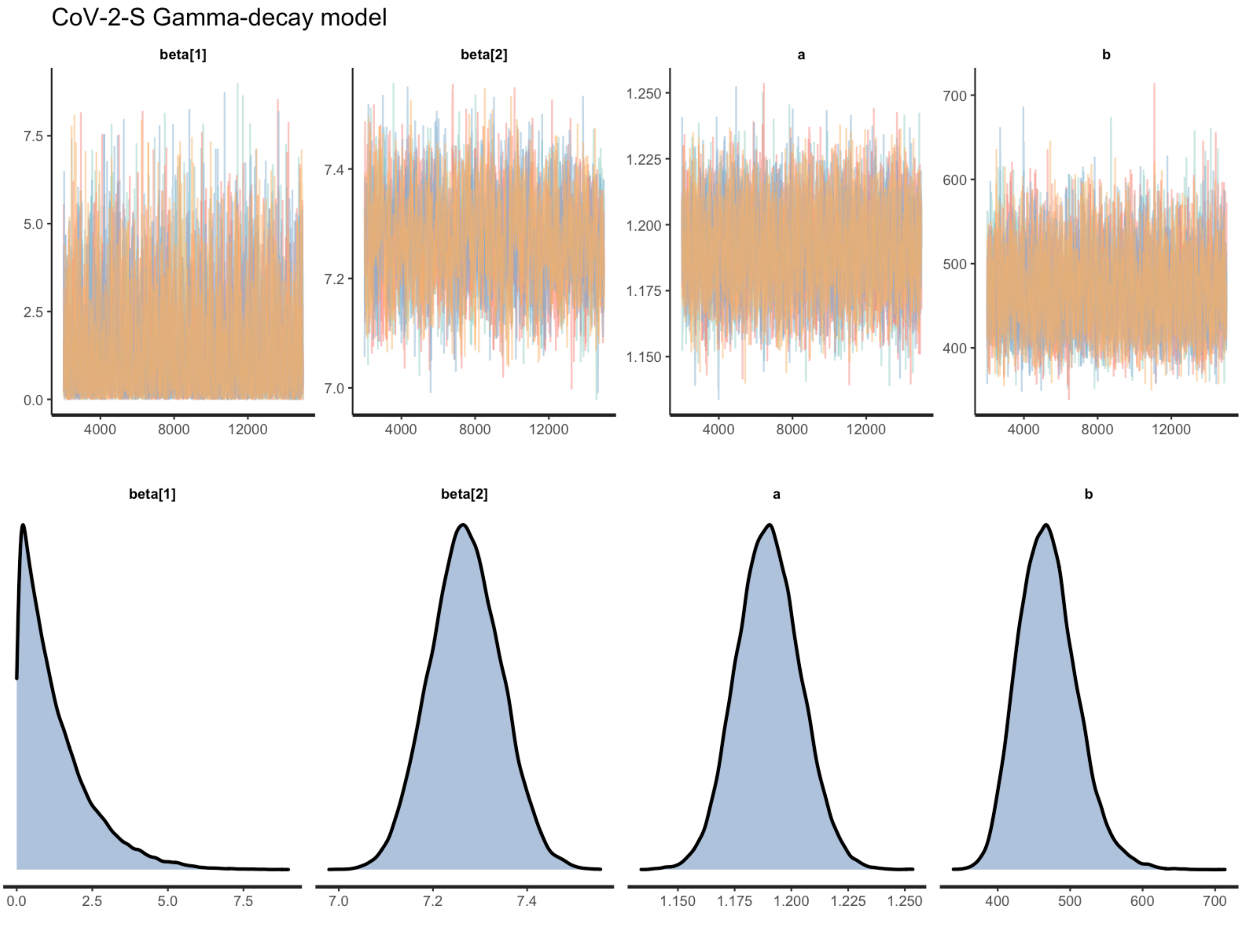
CoV-2-S Gamma-decay model
beta[1]
beta[2]
a
b
0.0
2.5
5.0
7.5
7.0
7.2
7.4
1.150
1.175
1.200
1.225
1.250
400
500
600
700
4000
8000
12000

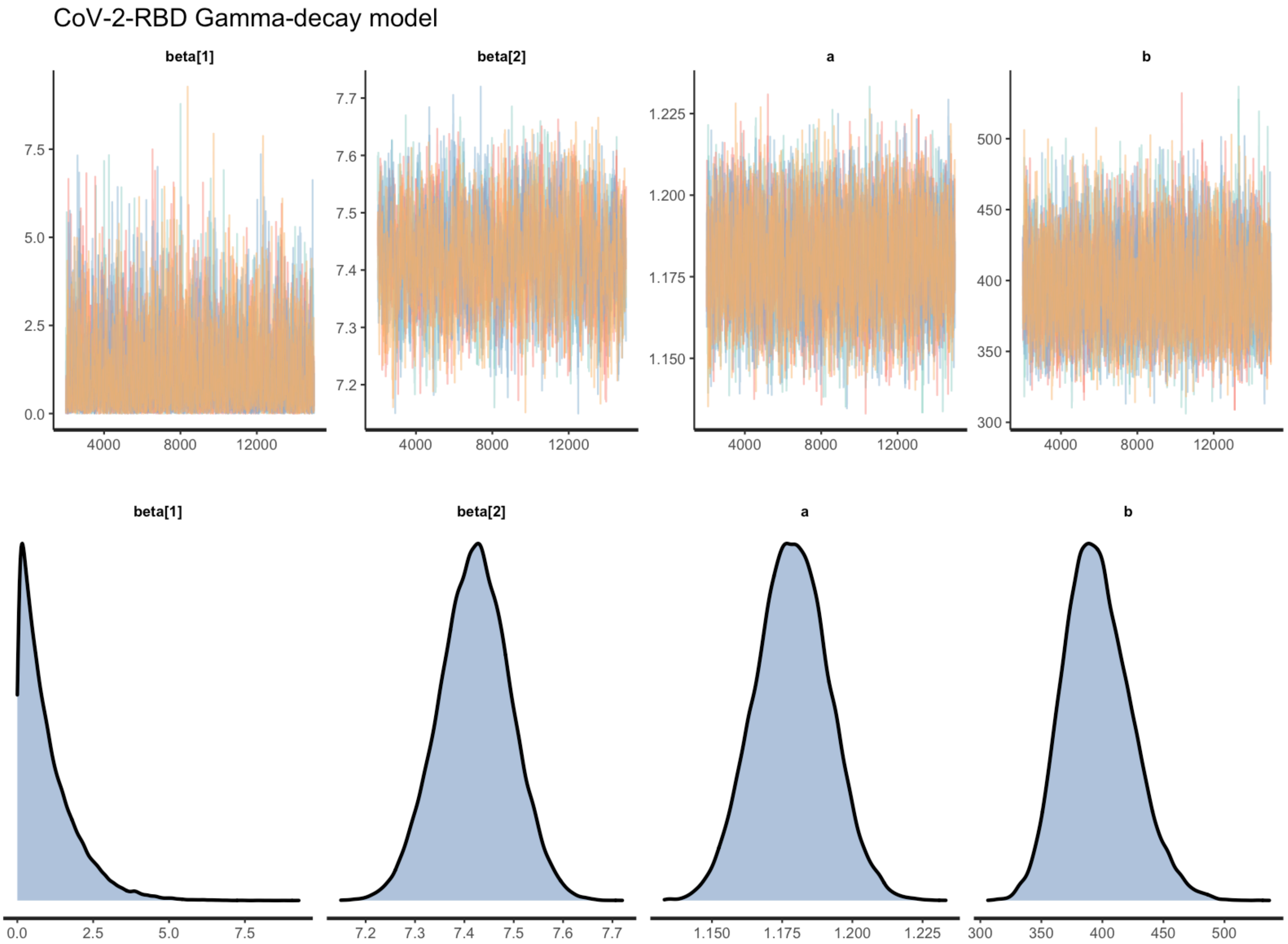

CoV-2-RBD Gamma-decay model
beta[1]
beta[2]
a
b
0.0
2.5
5.0
7.5
7.2
7.3
7.4
7.5
7.6
7.7
1.150
1.175
1.200
1.225
300
350
400
450
500
4000
8000
12000

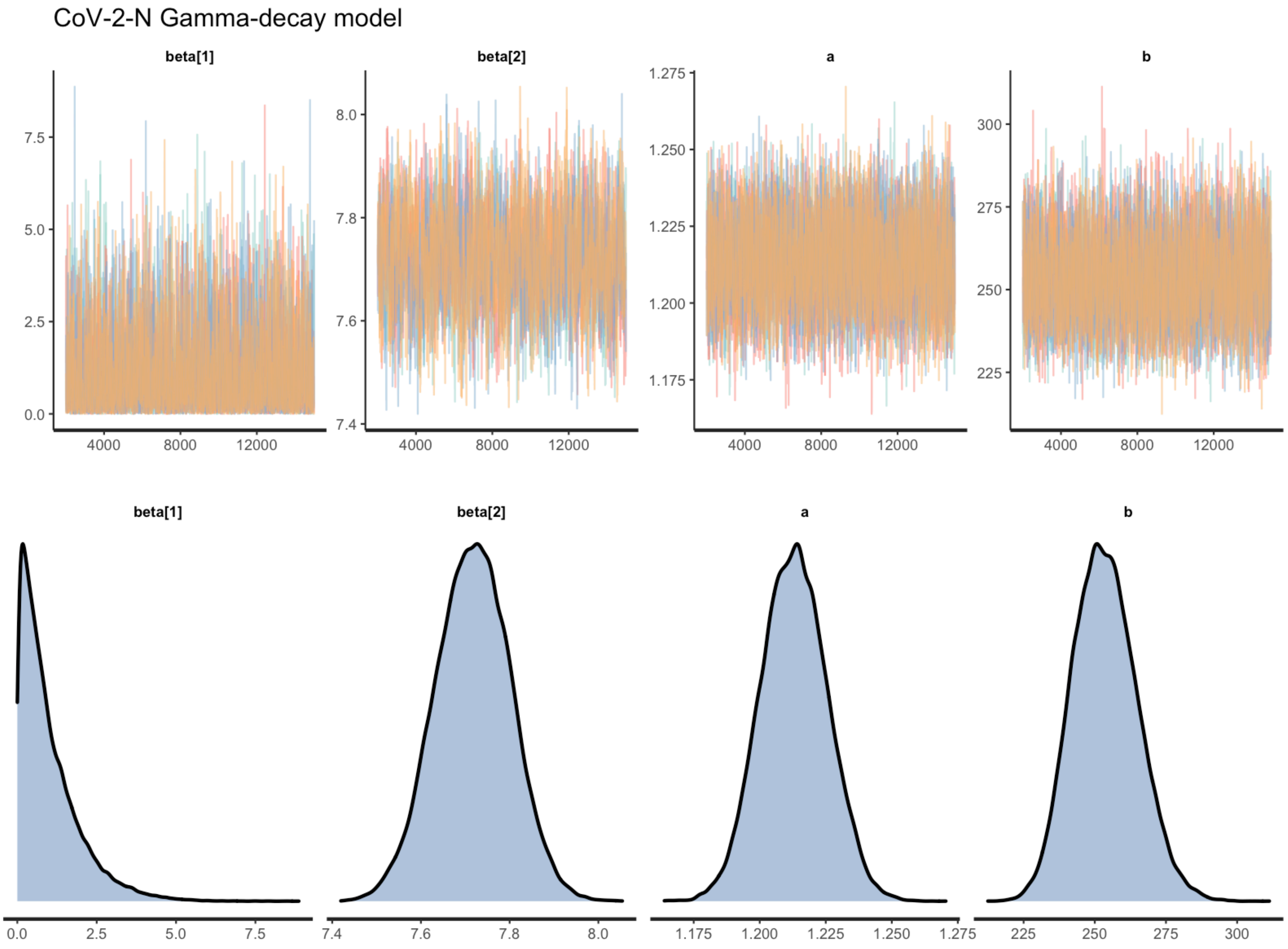

CoV-2-N Gamma-decay model
beta[1]
beta[2]
a
b
7.5
5.0
2.5
0.0
8.0
7.8
7.6
7.4
1.275
1.250
1.225
1.200
1.175
300
275
250
225
4000
8000
12000
beta[1]
beta[2]
a
b
0.0
2.5
5.0
7.5
7.4
7.6
7.8
8.0
1.175
1.200
1.225
1.250
1.275
225
250
275
300

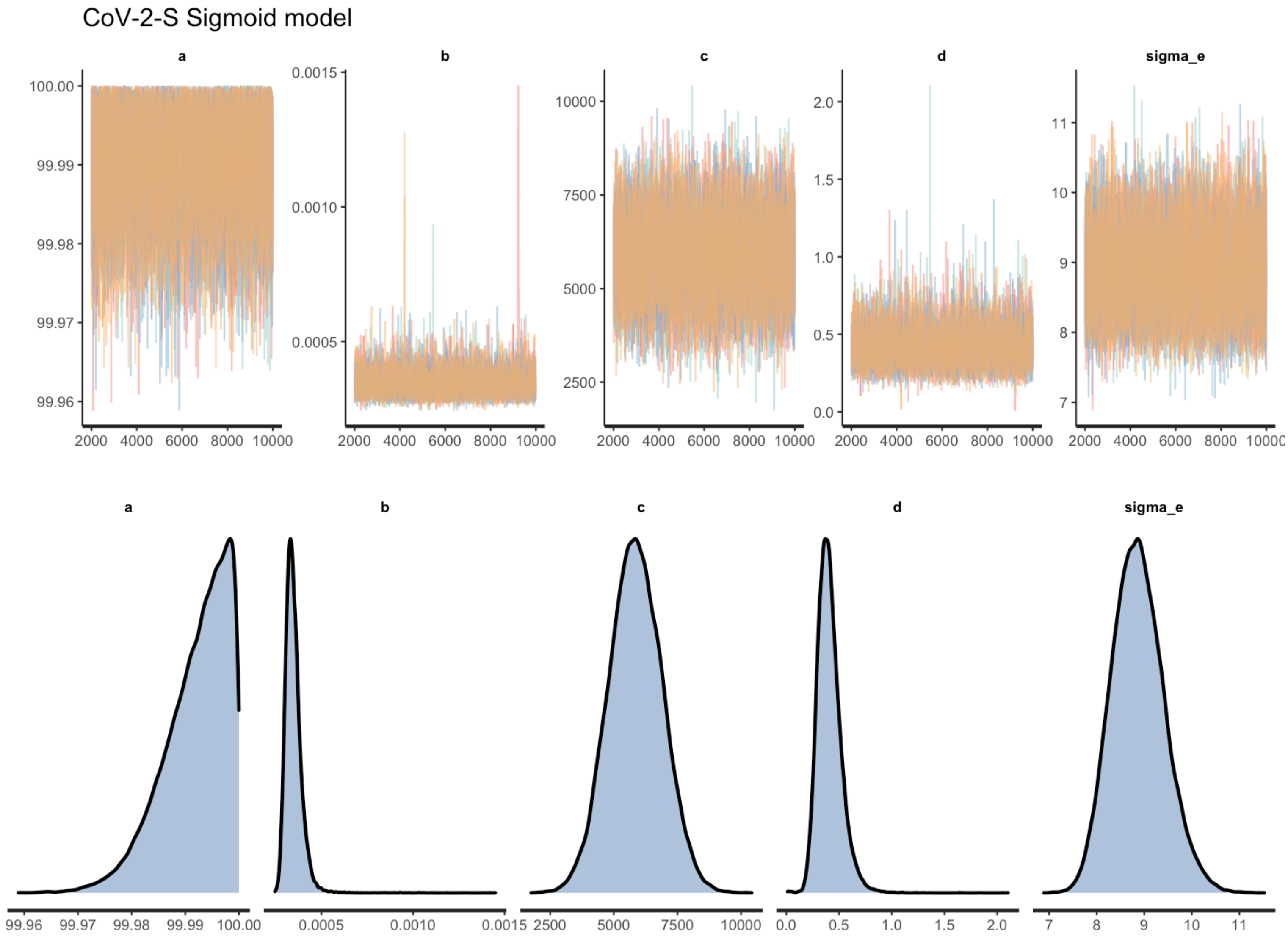

CoV-2-S Sigmoid model
a
b
c
d
sigma_e
100.00
99.99
99.98
99.97
99.96
0.0015
0.0010
0.0005
10000
7500
5000
2500
2.0
1.5
1.0
0.5
0.0
11
10
9
8
7
2000 4000 6000 8000 10000
a
b
c
d
sigma_e
99.96 99.97 99.98 99.99 100.00
0.0005 0.0010 0.0015
2500 5000 7500 10000
0.0 0.5 1.0 1.5 2.0
7 8 9 10 11

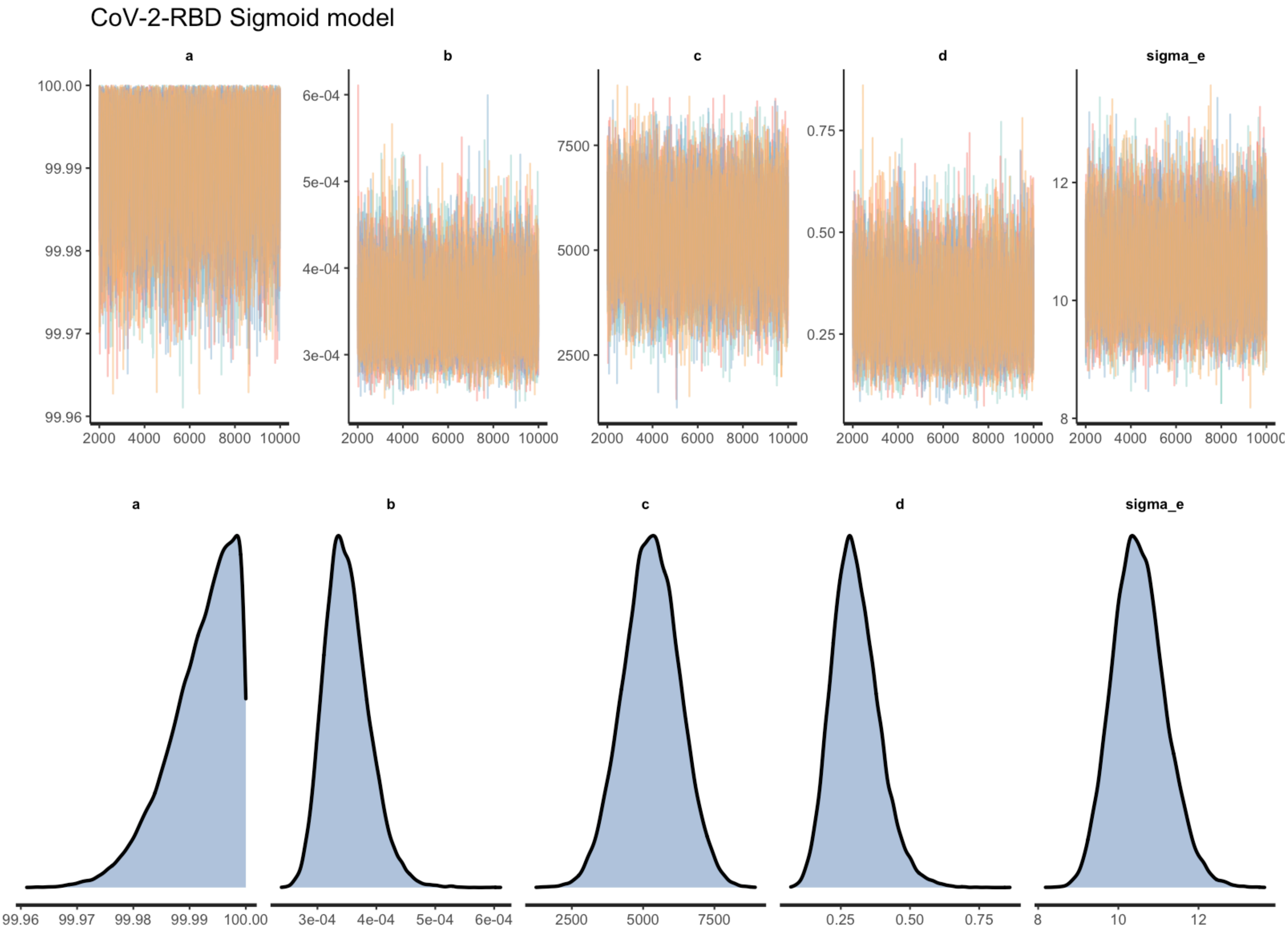

CoV-2-RBD Sigmoid model
a
b
c
d
sigma_e
100.00
99.99
99.98
99.97
99.96
2000
4000
6000
8000
10000
6e-04
5e-04
4e-04
3e-04
7500
5000
2500
0.75
0.50
0.25
12
10
8
99.96
99.97
99.98
99.99
100.00
3e-04
4e-04
5e-04
6e-04
2500
5000
7500
0.25
0.50
0.75
8
10
12
