# Supplementary material for: Long-Term Persistence of Spike Protein Antibody and Predictive Modeling of Antibody Dynamics After Infection With Severe Acute Respiratory Syndrome Coronavirus 2
Source: Clin Infect Dis. 2021 Jul 4;74(7):1220–9. doi: 10.1093/cid/ciab607 (PMC8994590; doi:10.1093/cid/ciab607)
Supplement: ciab607_suppl_Supplementary_Methods [file ciab607_suppl_supplementary_methods.docx]

**Supplementary Methods**

**Serological Test Validation and Antibody Titer Quantification:** Before the study took place a performance evaluation of the EDI^TM^ assay was undertaken using 47 PCR positive controls and 52 pre-2019 negative control serum samples. This established a sensitivity and specificity of 92% and 96% respectively for the EDI^TM^ assay. There was good concordance between the EDI^TM^ assay and the MSD assay, the EDI^TM^ assay detecting 94% of positive tests that the MSD assay detected among PCR positive samples >14 days post infection. MSD assay quantification and performance were evaluated as described in our accompanying methods paper [57]. Briefly, IgG levels for the MSD assay were expressed as arbitrary units calibrated against a set of reference sera distributed by the National Institute of Biological Standards and Control (Potters Bar, UK) under the auspices of the World Health Organisation. SARS-CoV-2 real-time reverse-transcriptase polymerase chain reaction (RT-PCR) targeting the N-gene was performed following RNA extraction, as previously described [61].

**Statistical Analysis and Power Calculations:** Power calculations were based on a negative exponential model of antibody decay from the peak using the pwr.f2.test function in R. We assumed a study power of 80% and explored a variety of hypothesized effect sizes (decreases in antibody titers over 1 year) and co-variates on the required study size with an alpha of 0.05 (Supplementary Materials, Study Protocol). When subjects did not return for repeated sampling, they were considered censored in our survival analysis. Loss to follow up during the study decreased the effective sample size as follow up time increased. Confidence limits of the observed time-to-negativity were implemented using the R-package “survival”.

**Statistical analysis and modeling of antibody dynamics:** To calculate the proportion of individuals that serorevert over the course of the study, we performed a survival analysis to account for censoring using the survival package in R. An “event” was defined as a persistent negative test after the first positive test, while positive tests were counted as “censored” events.

The dynamics of antibody response following infection with SARS-CoV-2 were estimated by fitting two mixed effects models based on a gamma curve on every participant that had more than three antibody observations. Two gamma models were chosen (“gamma-plateau” and “gamma-decay”) to enable modeling of an optimistic upper bound estimate (eventual stabilization of decay to a plateau) to a pessimistic lower bound estimate (continuous exponential decay to zero).

Gamma-decay and gamma-plateau models were fitted to the entire antibody response curve from day 0 (symptom onset) to the peak and then subsequent decay of each SARS-CoV-2 antibody. The confidence limits around the curves were derived by repeated sampling from the posterior distribution of the different model parameters, including individual effects.

The gamma-decay model assumed an uninterrupted continuous decay, given by the formula:


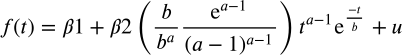


Eq1:

where f(t) is the log antibody titer at time t after symptom onset. The gamma function is described in terms of the shape (a) and scale (b), parameterized to reduce confounding of the parameters. β1 is the initial titer value at baseline; β2 determines the level of antibody rise; and u is an individual effect.

To account for the contribution of long-lived plasma cells, a third term was added to the model, allowing for a long-term plateau expressed as:


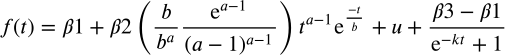


Eq2:

Where β3 represents the long-term plateau, and k is the rate at which the latter term rises.

The relationship between ACE-2 receptor blocking and antibody titers was modeled with a 4-parameter generalized logistic curve, where the percentage binding at titer level t is given by:


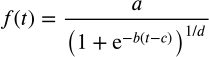


Eq3:

With parameters a,b, and c that represent, respectively, the upper receptor blocking asymptote, the growth rate, and the titer at which maximum growth occurs. The parameter d is an asymmetry factor that affects the point of inflection on the y axis.

All the models were fitted using RSTAN in R (R: A language and environment for statistical computing. Foundation for Statistical Computing, Vienna, Austria). For each model, we ran 4 independent chains for 15,000 iterations for the gamma distributions, and 10,000 iterations for the sigmoid model. Model comparison was performed using Pareto-smoothed importance sampling leave-one-out cross-validation (PSIS-LOO) as implemented in the loo R package. Monte-Carlo Markov traces converged well for both the gamma distribution and decay-to-plateau curves demonstrating a stable model fit to the data (Supplementary Figure 1). The maximum R-hat for any parameter was 1.0035, while the minimum effective sample size (ESS) was 842.8 (Supplementary data, Table S1).
