# Supplementary material for: Long-Term Persistence of Spike Protein Antibody and Predictive Modeling of Antibody Dynamics After Infection With Severe Acute Respiratory Syndrome Coronavirus 2
Source: Clin Infect Dis. 2021 Jul 4;74(7):1220–9. doi: 10.1093/cid/ciab607 (PMC8994590; doi:10.1093/cid/ciab607)
Supplement: ciab607_suppl_Supplementary_Tables [file ciab607_suppl_supplementary_tables.docx]

**Supplementary Table S1 - Posterior distribution estimates and model convergence summary**

| **Model** | **Antigen** | **Parameter** | **Mean** | **Median** | **2.5% CI** | **97.5% CI** | **ESS** | **R-hat** |
| --- | --- | --- | --- | --- | --- | --- | --- | --- |
| **Gamma-plateau** | **S** | **beta1** | 2.56 | 2.71 | 0.40 | 4.01 | 7290.13 | 1.00069 |
|  |  | **beta2** | 0.93 | 0.85 | 0.33 | 1.96 | 19735.40 | 0.99997 |
|  |  | **beta3** | 6.38 | 6.45 | 5.30 | 7.02 | 15862.35 | 1.00014 |
|  |  | **a** | 1.17 | 1.11 | 1.01 | 1.71 | 24185.03 | 1.00009 |
|  |  | **b** | 219.06 | 219.67 | 24.53 | 434.13 | 18160.67 | 1.00028 |
|  |  | **k** | 0.29 | 0.27 | 0.16 | 0.56 | 17365.52 | 1.00034 |
|  | **RBD** | **beta1** | 2.35 | 2.40 | 0.37 | 4.14 | 7850.09 | 1.00015 |
|  |  | **beta2** | 1.58 | 1.48 | 0.87 | 2.86 | 8768.42 | 1.00023 |
|  |  | **beta3** | 6.18 | 6.34 | 4.81 | 6.82 | 8764.88 | 1.00026 |
|  |  | **a** | 1.20 | 1.11 | 1.00 | 1.92 | 16136.09 | 1.00003 |
|  |  | **b** | 135.27 | 112.05 | 34.75 | 344.81 | 9007.35 | 1.00018 |
|  |  | **k** | 0.29 | 0.27 | 0.17 | 0.53 | 17691.86 | 1.00001 |
|  | **N** | **beta1** | 1.21 | 1.02 | 0.05 | 3.31 | 4898.48 | 1.00038 |
|  |  | **beta2** | 3.78 | 3.73 | 2.56 | 5.23 | 3144.69 | 1.00087 |
|  |  | **beta3** | 4.21 | 4.28 | 2.68 | 5.41 | 3289.29 | 1.00076 |
|  |  | **a** | 1.22 | 1.19 | 1.08 | 1.52 | 5808.56 | 1.00074 |
|  |  | **b** | 150.67 | 148.24 | 74.72 | 236.42 | 3335.84 | 1.00088 |
|  |  | **k** | 0.26 | 0.23 | 0.15 | 0.56 | 12731.13 | 1.00014 |
| **Gamma-decay** | **S** | **beta1** | 1.27 | 0.93 | 0.03 | 4.37 | 4746.78 | 1.00094 |
|  |  | **beta2** | 7.27 | 7.27 | 7.12 | 7.42 | 1142.77 | 1.00356 |
|  |  | **a** | 1.19 | 1.19 | 1.16 | 1.22 | 3218.21 | 1.00221 |
|  |  | **b** | 469.40 | 466.97 | 398.53 | 557.07 | 3230.69 | 1.00214 |
|  | **RBD** | **beta1** | 0.99 | 0.71 | 0.03 | 3.46 | 4213.02 | 1.00126 |
|  |  | **beta2** | 7.42 | 7.42 | 7.28 | 7.56 | 1042.43 | 1.00301 |
|  |  | **a** | 1.18 | 1.18 | 1.15 | 1.20 | 2729.89 | 1.00082 |
|  |  | **b** | 396.19 | 394.23 | 347.38 | 455.32 | 2735.57 | 1.00120 |
|  | **N** | **beta1** | 1.01 | 0.73 | 0.03 | 3.51 | 4786.50 | 1.00096 |
|  |  | **beta2** | 7.72 | 7.72 | 7.55 | 7.88 | 842.78 | 1.00572 |
|  |  | **a** | 1.21 | 1.21 | 1.19 | 1.24 | 3447.14 | 1.00217 |
|  |  | **b** | 253.60 | 253.15 | 232.89 | 276.65 | 3082.98 | 1.00252 |
| **Sigmoid** | **S** | **a** | 99.99 | 99.99 | 99.98 | 100.00 | 16995.30 | 1.00022 |
|  |  | **b** | 0.00 | 0.00 | 0.00 | 0.00 | 4278.59 | 1.00122 |
|  |  | **c** | 5854.63 | 5847.40 | 3829.62 | 7913.68 | 9890.67 | 1.00030 |
|  |  | **d** | 0.41 | 0.40 | 0.23 | 0.66 | 8331.01 | 1.00011 |
|  | **RBD** | **a** | 99.99 | 99.99 | 99.98 | 100.00 | 18844.79 | 1.00015 |
|  |  | **b** | 0.00 | 0.00 | 0.00 | 0.00 | 17737.25 | 1.00010 |
|  |  | **c** | 5286.62 | 5289.01 | 3354.71 | 7210.93 | 11869.92 | 1.00063 |
|  |  | **d** | 0.30 | 0.30 | 0.16 | 0.49 | 12695.78 | 1.00063 |

**Supplementary Table S2 - Model comparison summary.**

| **Antigen** | **Model** | **WAIC** | **WAIC SE** | **ELPD** | **ELPD SE** |
| --- | --- | --- | --- | --- | --- |
| **S** | Gamma plateau | -1117.24 | 46.56 | -1129.73 | 47.57 |
| **S** | Gamma decay | -1170.36 | 39.18 | -1182.48 | 40.22 |
| **RBD** | Gamma plateau | -987.59 | 43.31 | -1000.95 | 44.61 |
| **RBD** | Gamma decay | -1061.868 | 38.38 | -1071.83 | 39.06 |
| **N** | Gamma plateau | -983.15 | 50.55 | -994.85 | 51.26 |
| **N** | Gamma decay | -1017.95 | 47.07 | -1030.21 | 48.19 |

**Supplementary Figure Legends**

**Supplementary Figure 1 - Monte Carlo Markov Chain Plots for Model Fits.**

Attached as separate file
